# Supplementary material for: A Regulatory Loop Involving miR-200c and NF-κB Modulates Mortalin Expression and Increases Cisplatin Sensitivity in an Ovarian Cancer Cell Line Model
Source: Int J Mol Sci. 2022 Dec 4;23(23):15300. doi: 10.3390/ijms232315300 (PMC9737914; doi:10.3390/ijms232315300)
Supplement: Supplementary file 1 [file ijms-23-15300-s001.zip › ijms-1996186-supplementary.pdf]

Supplementary figures

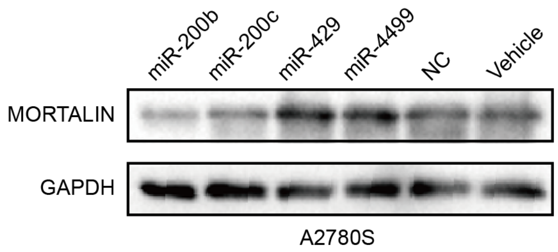

Supplementary Figure S1. MiR-429 does not inhibit the expression of mortalin in ovarian cancer cells.

Western blot was used to detect the protein expression of mortalin in ovarian cancer cells after transfection of miRNAs mimic.

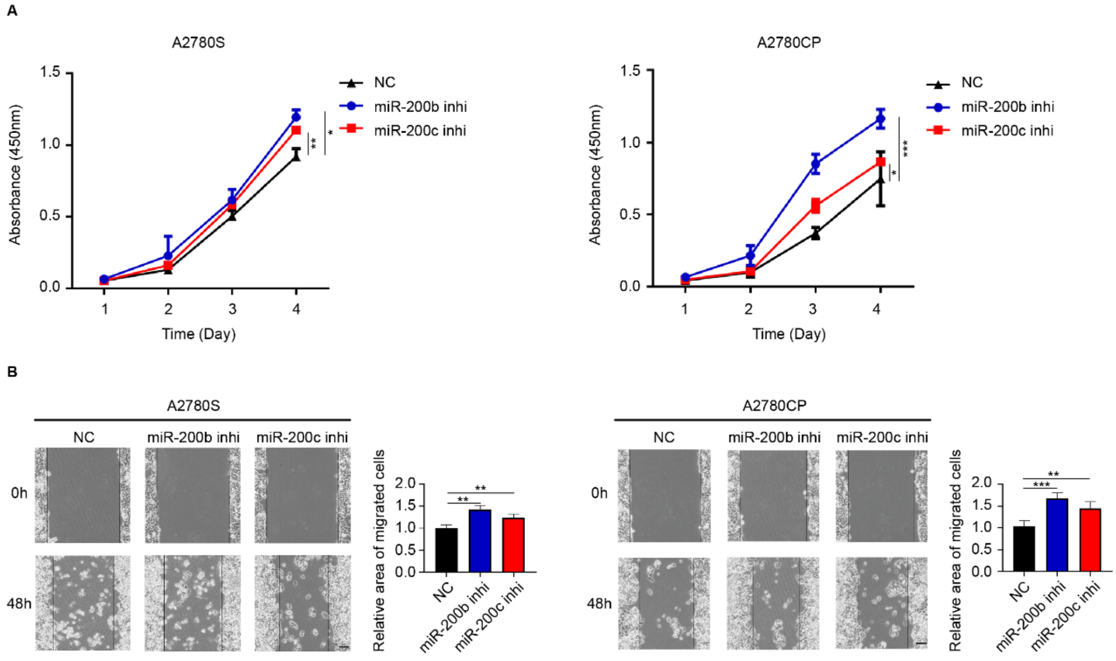

Supplementary Figure S2. Inhibition of miR-200b/c increases proliferation and migration of ovarian cancer cells.

After transfection of miR-200b/c inhibitor or NC to A2780S (left) and A2780CP (right) cells, (A) the cell viability was detected using CCK-8 assay. (B) The migration of ovarian cancer cells was detected by wound healing assay (scale bar 20 μm). Biological replication, n=3. **P*<0.05, ***P*<0.01, ****P*<0.001.

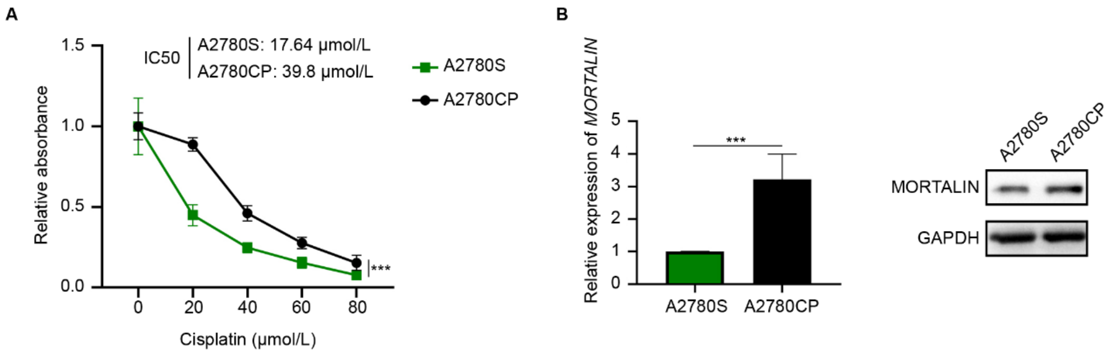

Supplementary Figure S3. The expression of mortalin is positively correlated with cisplatin resistance.

(A) Different concentrations of cisplatin were applied to ovarian cancer cell lines A2780S and A2780CP for 24 hours, and the cell viability was detected by CCK-8 assay. (B) The mRNA (left) and protein (right) expression of mortalin were detected by qPCR and western blot. n=3. $^{***}P<0.001$.

Supplementary Table S1. qPCR primers for various genes used in this study.

Gene	Primer
GAPDH	F：5’-GACCTGACCTGCCGTCTAG-3’
	R：5’-AGGAGTGGGTGTCGCTGT-3’
MORTALIN	F: 5'-TGGTGAGCGACTTGTTGGAAT-3
	R: 5'-ATTGGAGGCACGGACAATTTT-3'
NF-κB p65	F：5’-ATGTGGAGATCATTGAGCAGC-3’
	R: 5’-CCTGGTCCTGTGTAGCCATT-3’
MIR-200B promoter	F: 5’-TGCTGTCCAGGCCTTCCTATGGGA-3’
	R: 5’-TTACCGAAGTGTCCTGGCCAGACG-3’
PTPN6/MIR-200C promoter	F: 5’-TTCTCTGAGGAACTGGGCTGTTAG-3’
	R: 5’- GGTAGGGGAACAGACCAGGAAGTAA-3’
